# Source Analysis of Ozone Pollution in Liaoyuan City’s Atmosphere Based on Machine Learning Models and HYSPLIT Clustering Method

**DOI:** 10.3390/toxics13060500

**Published:** 2025-06-13

**Authors:** Xinyu Zou, Xinlong Li, Dali Wang, Ju Wang

**Affiliations:** 1 College of New Energy and Environment, Jilin University, Changchun 130012, China; zouxy9922@mails.jlu.edu.cn (X.Z.); xinlong22@mails.jlu.edu.cn (X.L.); 2 Environmental Sciences Division, Oak Ridge National Laboratory, Oak Ridge, TN 37831, USA; wangd@ornl.gov; 3 Key Laboratory of Groundwater Resources and Environment, Ministry of Education, Jilin University, Changchun 130021, China; 4 Jilin Province Key Laboratory of Water Resources and Environment, Jilin University, Changchun 130021, China

**Keywords:** ozone pollution, machine learning, random forest, regional transport, HYSPLIT clustering

## Abstract

Firstly, this study investigates the spatiotemporal distribution characteristics of the ozone (O_3_) pollution in Liaoyuan City using monitoring data from 2015 to 2024. Then, three machine learning models (ML)—random forest (RF), support vector machine (SVM), and artificial neural network (ANN)—are employed to quantify the influence of meteorological and non-meteorological factors on O_3_ concentrations. Finally, the HYSPLIT clustering method and CMAQ model are utilized to analyze inter-regional transport characteristics, identifying the causes of O_3_ pollution. The results indicate that O_3_ pollution in Liaoyuan exhibits a distinct seasonal pattern, with the highest concentrations found in spring and summer, peaking in the afternoon. Among the three ML models, the random forest model demonstrates the best predictive performance (R^2^ = 0.9043). Feature importance identifies NO_2_ as the primary driving factor, followed by meteorological conditions in the second quarter and land surface characteristics. Furthermore, regional transport significantly contributes to O_3_ pollution, with approximately 80% of air mass trajectories in heavily polluted episodes originating from adjacent industrial areas and the sea. The combined effects of transboundary precursors and O_3_ transport with local emissions and meteorological conditions further increase the O_3_ pollution level. This study highlights the need to strengthen coordinated NO_X_ and VOCs emission reductions and enhance regional joint prevention and control strategies in China.

## 1. Introduction

Ground-level ozone is an important secondary pollutant, and its accumulation is primarily driven by two precursors: volatile organic compounds (VOCs) and nitrogen oxides (NO_X_) [1]. These undergo photochemical reactions under sunlight to produce ozone. When ozone concentration exceeds a certain level, it will cause harmful effects for human health, the environment, etc. VOCs originate from both anthropogenic and natural sources, while NO_X_ is mainly emitted from combustion processes and natural sources. Meanwhile, as one of the key volatile organic compounds, isoprene plays a crucial role in the formation of tropospheric O_3_ [2,3]. However, the increase in ground-level O_3_ concentrations is not solely caused by the increase in anthropogenic and natural emissions but is also significantly influenced by meteorological conditions and temporal factors [4]. Moreover, the relationship between meteorological drivers and O_3_ concentrations varies across regions [5].

In recent years, machine learning (ML) methods have gained widespread application and recognition in the field of atmospheric pollution prediction [6,7,8]. ML has been proven effective for predicting O_3_ concentration trends, as it can capture spatial and temporal details while reducing variance and errors in high-dimensional datasets. Many studies that employed ML to investigate the causes of O_3_ have yielded insightful findings. Consequently, ML algorithms are frequently applied to study the driving factors of ground-level O_3_ concentrations [9,10]. For instance, Yang et al. [11] employed an RF model to study O_3_ pollution in the Sichuan Basin from 2017 to 2020. Their results indicate that the O_3_ prediction model constructed based on RF has a high goodness of fit, demonstrating excellent stability and generalization ability. Except for Ya’an, the variable interpretation rates of all the prediction models for other cities reached over 80%. Huang et al. [12] use an RF model to demonstrate that the top three most significant influencing factors on O_3_ concentrations are NO_2_, RH, and T. O_3_ concentrations have a strong linear relationship with RH and T and a strong nonlinear relationship with NO_2_. Aziz et al. [13] find that the ANN model could provide reliable predictions of regional O_3_ concentrations for the following day and also emphasizes the significance of meteorological factors and emission patterns in influencing the concentration of O_3_. Su et al. [14] utilize the SVM method to analyze the meteorological and observational data of O_3_ and its precursors. Their research shows that compared with the multiple linear regression method, SVM has obvious advantages in predicting O_3_ concentration. Studies conducted in Malaysia [15], India [16], and Brazil [17] have compared multiple ML methods, and their results indicate that the RF, SVM, and ANN models exhibit superior performance in predicting ground-level O_3_ concentrations.

The HYSPLIT model can be used to track the influence of regional transport on ground-level O_3_ and identify the sources of ground-level O_3_. Lin et al. [18] utilize the HYSPLIT model to establish backward trajectory, investigating the causes and sources of O_3_ pollution in Rizhao City over the summer of 2020. The results reveal that among all the paths, the path from the west of Rizhao City, accounting for the largest proportion of exceedances, is the main transportation channel for O_3_ and its precursors. Zhang et al. [19] employed the HYSPLIT model to study the causes of high O_3_ pollution in the suburbs of Shanghai in July 2016. Their results indicate that Zhejiang Province is the primary potential source of O_3_ in Shanghai’s suburban areas. When the study area is dominated by southwesterly winds exceeding 2 m/s, O_3_-enriched air masses from upwind regions could be transported to Shanghai’s suburbs. This study highlights the significant influence of regional meteorological conditions and pollution source distribution on O_3_ transport, providing a reference for analyzing the regional transport factors of O_3_ pollution in Liaoyuan City. It also suggests that Liaoyuan City might similarly be affected by pollution source transport from surrounding regions.

Liaoyuan City is located in the central–southern part of Jilin Province, covering 2.8% of the province’s total area, making it the smallest prefecture-level city in Jilin by size. Situated in the transitional zone between the Changbai Mountain foothills and the Songliao Plain, the local terrain gradually slopes from the southeast to the northwest. The average altitude is 300 m, and the urban area features diverse terrains dominated by low mountains, hills, and plains. The climate is characterized by semi-humid temperate continental monsoon conditions, with distinct seasons. Summers are warm and rainy, while winters are cold, dry, and prolonged. The annual average temperature is approximately 5.2 °C, and the annual precipitation ranges between 600 and 700 mm, with over 60% of rainfall occurring in the summer months (June to August). From 2019 to 2023, Liaoyuan City’s gross domestic product (GDP) increased year by year, reaching CNY 51.688 billion in 2023. The tertiary sector contributed 71.7% to the GDP, becoming the primary driver of economic growth. In recent years, Liaoyuan City has experienced significant population outflow, with a distribution pattern characterized by “central urban concentration and county-level dispersion.” By the end of 2023, the city’s permanent population was 943,800, with an urbanization rate of approximately 60%. As a part of the old industrial base of Northeast China, Liaoyuan City has faced environmental challenges such as air pollution and the management of coal mining subsidence areas. In recent years, through industrial upgrading and environmental remediation, the proportion of days with excellent air quality reached 89.6% in 2024. However, summer O_3_ pollution has become increasingly prominent, making Liaoyuan a hotspot for such pollution in Jilin Province. Therefore, this study utilizes machine learning, WRF-CMAQ, and regional transport analysis methods to explore the primary causes of O_3_ pollution, providing a scientific basis for regional air pollution control.

## 2. Methods and Data

### 2.1. Random Forest Model

RF is a machine learning algorithm based on ensemble learning [20]. Its core principle is enhancing the model’s generalization ability and stability through a collaborative decision-making mechanism involving multiple decision trees. The algorithm employs the Bootstrap resampling technique to draw multiple subsamples with replacements from the original dataset, with each subsample being the same size as the original dataset [21]. Each subsample is independently used to train a decision tree. When generating a single decision tree, only a randomly selected subset of features is considered for optimal splitting at each node. This randomness in feature selection reduces the correlation among different decision trees, thereby effectively mitigating the risk of overfitting [22]. The final prediction is determined by either the voting of different decision trees in a classification task or the mean of their predictions in a regression task.

### 2.2. Support Vector Machine Model

SVM is a supervised learning model based on statistical learning theory [23]. Its core principle is achieving optimal classification or regression by constructing a hyperplane with a maximum geometric margin [24]. The theoretical framework of SVM is grounded in structural risk minimization [25], which optimizes generalization performance by balancing training error and model complexity. Additionally, since the selection of support vectors relies only on the sample points near the classification boundary, the model exhibits a strong robustness to noisy data points that are far from the boundary.

### 2.3. Artificial Neural Network Model

ANN is a computational model inspired by biological neural systems [26]. It constructs a multi-layered computational structure by simulating biological neural networks, including an input layer, hidden layers, and an output layer. The core mechanisms of ANN are forward propagation and back propagation. Research has shown that a neural network with a single hidden layer can approximate any continuous function, while deep neural networks, through hierarchical feature extraction, can reduce parameter complexity exponentially [27], thereby representing complex patterns more efficiently.

### 2.4. HYSPLIT Backward Trajectory Cluster Analysis

HYSPLIT (https://www.arl.noaa.gov/hysplit/ (accessed on 11 June 2025)) backward trajectory cluster analysis reveals commonalities in air mass transport pathways by combining meteorological models with clustering algorithms [28]. This approach is divided into two stages: trajectory calculation and cluster analysis. First, based on the Lagrangian particle dispersion model, the three-dimensional backward trajectories of air masses are calculated using meteorological reanalysis data, with the outputs being time-series spatial coordinates. Then, the similarity of the trajectory shapes is quantified through distance metrics. Finally, clustering algorithms are applied to group the trajectories into several clusters [29].

### 2.5. Simulation of O_3_ and Its Validation

**Figure 1:**
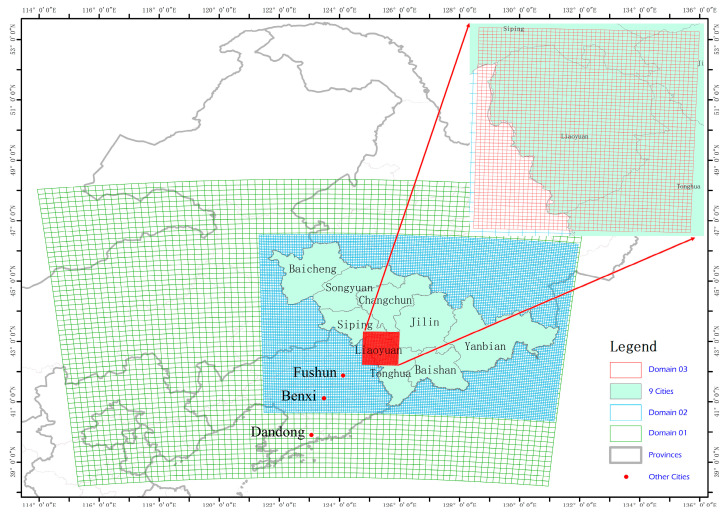
Three-layer nested map for WRF simulation.

To study the relationship between VOCs and O_3_ pollution in Liaoyuan City, especially the concentration distribution of isoprene, we use WRF 4.4.1 and CMAQ 5.4.0 to carry out air pollution simulation. The simulation period is from 15 June to 23 June, 2024, and we use the Lambert projection coordinate system. The central longitude and latitude of the study area are 43.431° N and 123.132° E. Three nested domains are set in the simulation, as shown in Figure 1. Among them, the first layer covers the northeastern region of China, with a grid resolution of 27 × 27 km and a grid number of 79 × 64. The second layer covers Jilin Province, with a grid resolution of 9 × 9 km and a grid number of 139 × 112. The third layer is Liaoyuan City, with a grid resolution of 3 × 3 km and a grid number of 52 × 64. The parameterization scheme used by the model is shown in Table 1. The statistical indicators for evaluating the simulation performance include the correlation coefficient R, mean fractional bias (MFB), and mean fractional error (MFE), shown in Table 2.

### 2.6. Data Sources

This study primarily utilizes three types of data, namely atmospheric environmental quality monitoring data, meteorological data, and major pollution source emission data, for Liaoyuan City. The atmospheric environmental quality monitoring data are obtained from the official website of the China National Environmental Monitoring Centre (http://www.cnemc.cn/ (accessed on 11 June 2025)), which includes hourly mass concentrations of six major pollutants from 1 January 2015 to 31 December 2024, collected at two automatic air quality monitoring stations in the city. The meteorological data are derived from the WRF v4.4.1 model (https://www.mmm.ucar.edu/models/wrf (accessed on 11 June 2025)) [30] developed by the National Center for Atmospheric Research (NCAR) in the United States, providing hourly data for 54 meteorological parameters in 2024. The major pollution source emission data are sourced from the Multi-resolution Emission Inventory for China (MEIC) (http://meicmodel.org.cn (accessed on 11 June 2025)) [31], developed by Tsinghua University and the Pollution Control Division of the Liaoyuan Municipal Ecological Environment Bureau.

## 3. Results and Discussion

### 3.1. Analysis of Atmospheric Pollution Characteristics in Liaoyuan City

#### 3.1.1. Analysis of Spatial Distribution Characteristics

**Figure 2:**
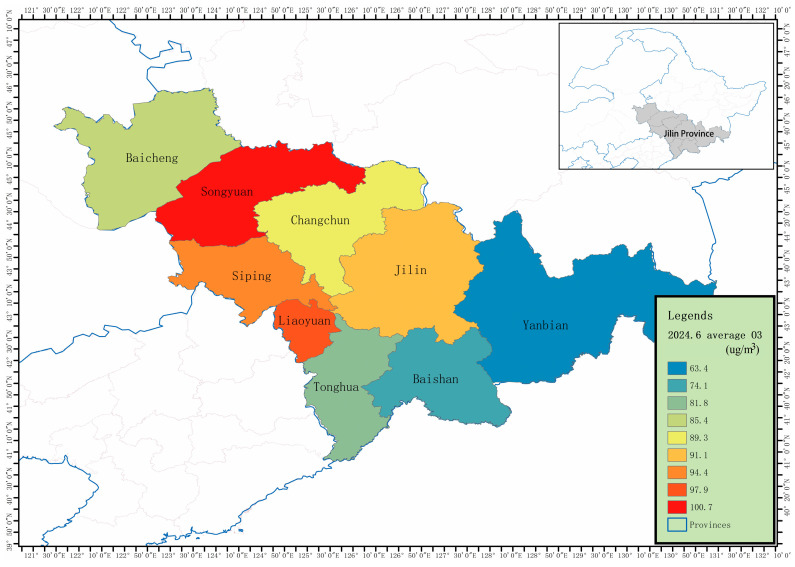
Geographic location of Jilin Province and ranking of average O_3_ pollution concentrations in June 2024.

Jilin Province comprises nine prefecture-level divisions: Changchun, Jilin, Siping, Liaoyuan, Tonghua, Baishan, Songyuan, Baicheng, and the Yanbian Korean Autonomous Prefecture. Figure 2 shows the ranking of the average O_3_ pollution concentrations in these nine regions for June 2024. As illustrated in this Figure, there are significant differences in the average O_3_ pollution concentrations among the regions in June. Spatially, the central and western parts of Jilin Province, such as Songyuan and Liaoyuan City, exhibit relatively higher pollution levels, while the eastern regions, including Yanbian Prefecture, show lower pollution levels. This reflects the spatial heterogeneity of O_3_ pollution across Jilin Province. Among the regions, Songyuan and Liaoyuan City have the highest pollution levels, whereas the Yanbian Korean Autonomous Prefecture has the lowest level.

#### 3.1.2. Correlation Analysis with Meteorological Factors

To investigate the relationship between atmospheric pollutants and meteorological factors, Table 3 presents the correlations between the concentrations of six atmospheric pollutants in Liaoyuan City in 2024 and local temperature (T), pressure (P), and wind speed (WS). The degree of correlation is expressed using the Pearson correlation coefficient (r). The results indicate that O_3_ exhibits negative correlations with the concentrations of the other five pollutants [32], with the strongest negative correlation observed between O_3_ and NO_2_ (r = −0.54). As is well known, O_3_ is not a primary emitted pollutant; NO_2_ and VOCs are its key precursors, and they undergo nonlinear reactions [33], forming a cyclic chain reaction process under sunlight. Temperature shows a significant positive correlation with O_3_ concentration (r = 0.21) [34], consistent with most research findings, as solar radiation serves as an energy source, providing the necessary conditions for photochemical reactions [35]. As radiation intensity increases, the photochemical reaction process accelerates, increasing O_3_ concentrations [36]. Atmospheric pressure also shows a significant positive correlation with O_3_ concentration (r = 0.23), likely because high pressure brings stable meteorological conditions that promote photochemical reactions and O_3_ accumulation [34].

#### 3.1.3. Annual Variation Characteristics of Pollution

According to the Technical Regulation on Ambient Air Quality Index (on trial) (HJ 633-2012), the proportions of the primary air pollutants in Liaoyuan City from 2021 to 2024 were calculated and are shown in Table 4. During this period, the primary pollutants only include O_3_, PM_10_, and PM_2_._5_. Among them, PM_10_ is not always the primary pollutant each year, and it accounts for a relatively small proportion. The proportion of PM_2.5_ as the primary pollutant fluctuates over the four years. Meanwhile, the proportion of days with O_3_ as the primary air pollutant out of the total exceedance days shows a year-to-year increasing trend, increasing from 31.03% in 2021 to 52.60% in 2024. This indicates that O_3_ pollution became increasingly severe over those four years and requires our attention.

#### 3.1.4. Seasonal Variation Characteristics of Pollution

**Figure 3:**
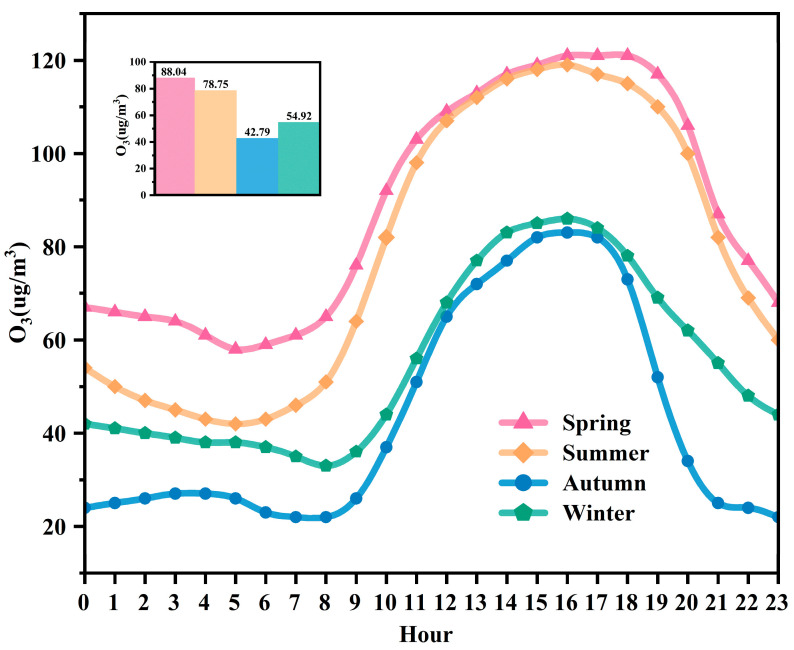
Daily variations in O_3_ concentrations in different seasons for 2024.

Figure 3 illustrates the seasonal variation in the O_3_ concentrations in Liaoyuan City in 2024. Overall, the O_3_ concentrations exhibit significant seasonal differences, with the most severe pollution occurring in spring, followed by summer, while autumn and winter show notably lower pollution levels compared to the other seasons. The average concentration difference between spring and autumn is 45.25 μg/m^3^. The diurnal variation trend of O_3_ in each season follows a single-peak and single-valley pattern, with daily peaks occurring between 14:00 and 17:00. The daily low values occur between 4:00 and 6:00 in spring and summer and between 7:00 and 9:00 in autumn and winter. This pattern can be attributed to the titration effect of NO on O_3_ during the early morning hours [37], maintaining O_3_ concentrations at low levels. In the morning, the accumulated NO_2_ from the previous night and the NO emissions from sources such as motor vehicles during the morning rush hour remain at high concentrations [38]. As the boundary layer height rises and solar radiation intensity significantly increases, the O_3_ generation mechanism dominated by photolysis reactions is rapidly activated [39], causing O_3_ concentrations to continuously increase and reach their peak at around 14:00 to 17:00.

#### 3.1.5. Monthly Variation Characteristics of Pollution

**Figure 4:**
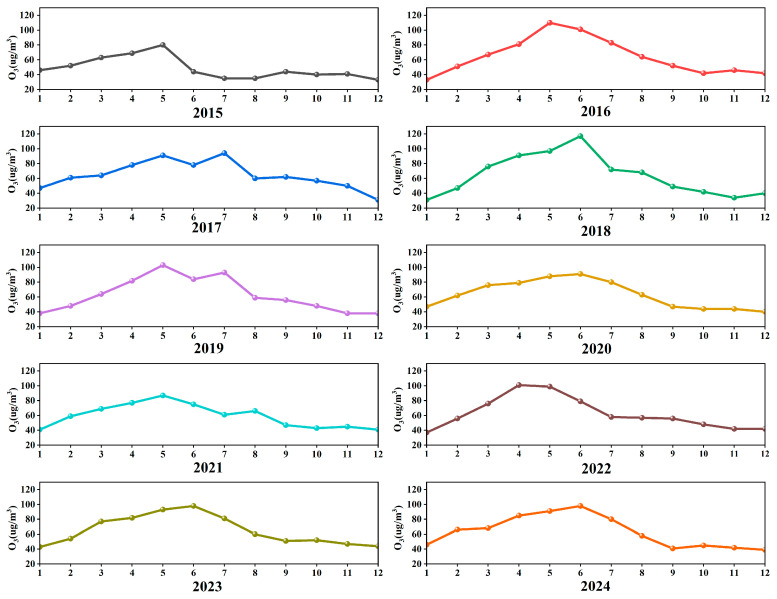
Monthly variations in O_3_ concentrations in Liaoyuan City from 2015 to 2024.

**Figure 5:**
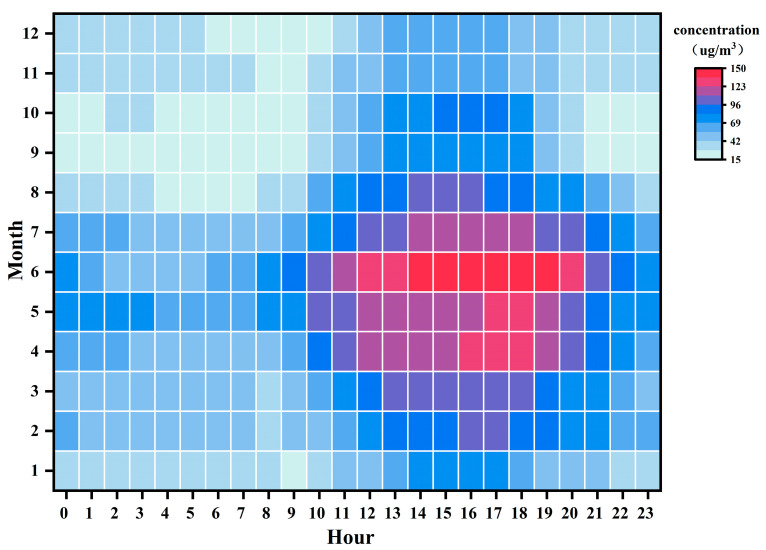
Hourly variations in O_3_ concentrations in different months for 2024.

Figure 4 shows the monthly average O_3_ concentration changes in Liaoyuan City from 2015 to 2024. Overall, the O_3_ concentrations exhibited significant monthly fluctuations, with their peak values concentrated in May and June. The highest monthly average concentration in the past decade was recorded in June 2018 (117 μg/m^3^). This phenomenon is closely related to the meteorological conditions in late spring and early summer, where increased sunshine duration and enhanced solar radiation accelerate photochemical reactions, while stable atmospheric conditions hinder pollutant dispersion [40]. Notably, abnormally low values were observed in 2020 and 2021, primarily due to the reduced anthropogenic emissions of O_3_ precursors during the COVID-19 pandemic [41]. Figure 5 illustrates the monthly variation in the O_3_ concentrations in Liaoyuan City in 2024. Contrasting with recent years, the O_3_ concentrations are higher from April to July in the afternoon to early evening periods, corresponding to the spring and summer seasons, closely associated with strong sunlight and higher temperatures [34]. Among these months, June experienced the most severe O_3_ pollution.

### 3.2. Factors Affecting Ozone Concentrations

#### 3.2.1. Comparative Study of Machine Learning Models

To thoroughly analyze the complex nonlinear relationships between the meteorological factors, non-meteorological factors, and O_3_ concentrations in Liaoyuan City, we decouple factors such as temporal variations, meteorological conditions, and pollution source emissions to investigate the specific contributions of these factors to O_3_ pollution. RF, ANN, and SVM, three models with strong nonlinear fitting capabilities, are selected for comparative study. The dataset used in this study covers hourly data for the entirety of 2024, divided into training and testing sets at a ratio of 3:1. Specifically, 6588 data points from 2024 are used for model training, while the remaining 2196 data points are used for model validation. To further improve computational efficiency, all data except temporal variables and wind direction are normalized before being input into the models. Additionally, the main parameters of the models are fine-tuned using a grid search method. The performances of the three models are comprehensively and accurately evaluated using metrics such as the coefficient of determination (R^2^), root mean square error (RMSE), and mean absolute error (MAE), combined with a five-fold cross-validation approach, to identify the best-performing parameterized model.

**Figure 6:**
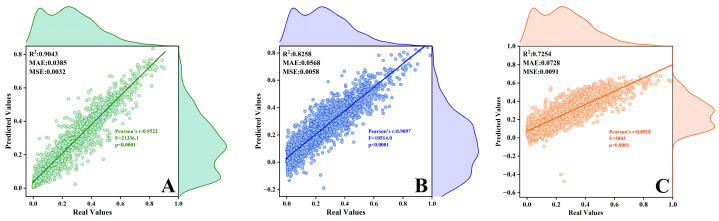
Regression fitting lines for O_3_ concentration using three machine learning methods in Liaoyuan City: (**A**) RF, (**B**) ANN, and (**C**) SVM.

Figure 6 presents the regression plots of the predicted versus actual values for the following three models: RF, ANN, and SVM. The detailed process and the other result of ML are shown in Figures S1–S3. In terms of evaluation metrics, the RF model demonstrates superior performance, achieving the highest R^2^ value of 0.9043, indicating the strongest ability to explain data variability. Compared to the other two models, RF exhibits the best goodness of fit to the data as well. Additionally, its MAE of 0.0385 and RMSE of 0.0032 are the lowest among the three models, suggesting that the smallest deviation between the predicted and actual values and the highest prediction accuracy are recorded using this method. In contrast, both ANN and SVM produce negative predictions for O_3_ concentrations in some cases, and their R^2^ values are lower than those of the RF model. In reality, O_3_ concentrations cannot be negative, and these prediction results highlight the limitations of ANN and SVM in handling the dataset and making predictions in this case. At the same time, we also perform linear fitting on the real values and predicted values and conduct a significance test. The results indicate that random forest has the best fitting effect. Overall, among the three ML models compared, the RF model performed the best in terms of overall simulation effectiveness; therefore, it is more accurate in quantifying the relationships between meteorological factors, non-meteorological factors, and O_3_ concentrations.

Based on the RF model, a systematic assessment of the factors influencing the ground-level ozone in Liaoyuan City is conducted. Through feature importance analysis, the synergistic mechanisms of multi-source driving factors are revealed. As shown in Table 5, the top 15 key independent variables ranked by their contribution are as follows: NO_2_ > Second Quarter > Vegetation Coverage > Sea Surface Temperature > PM_2.5_ > Water Vapor Content > Latent Heat > Downward Shortwave Radiation > CO > 10 m Wind Speed > 2 m Specific Humidity > Sensible Heat Flux > Ground Heat Flux > Surface Longwave Radiation > Outgoing Longwave Radiation. The results indicate that O_3_ generation is jointly regulated by precursor concentrations, meteorological factors, and underlying surface characteristics, with significant nonlinear interactions among different factors [37,42]. Among these, NO_2_ exhibits a significantly higher contribution than the other variables, serving as the primary driving factor. This underscores the high sensitivity of O_3_ generation to precursors [43]. The high contribution of the second quarter (April, May, and June) aligns well with the monthly peak O_3_ concentrations in Liaoyuan City. During this period, increased solar radiation, elevated boundary layer height, and high temperatures significantly and collectively create an optimal environment for photochemical reactions [44,45]. These meteorological conditions favor the generation and accumulation of O_3_, leading to peak concentrations occurring during this time. The 10 m wind speed and specific humidity reflect the inhibitory effects of atmospheric diffusion capacity and humidity conditions on O_3_ accumulation. The indirect regulatory role of underlying surface characteristics is manifested through vegetation coverage and surface energy fluxes (sensible heat, latent heat). This association with vegetation coverage may stem from the dual effects of volatile organic compounds released by plants participating in photochemical reactions [46]. Notably, although the emission data of the main pollution sources serving as independent variables do not rank among the top 15, their fundamental influence on precursor concentrations remains existent [37]. The model results might be limited by the insufficient spatiotemporal resolution of the emission data or collinearity with other variables, which could explain their absence in the top rankings.

#### 3.2.2. Analysis of Regional Transport

**Figure 7:**
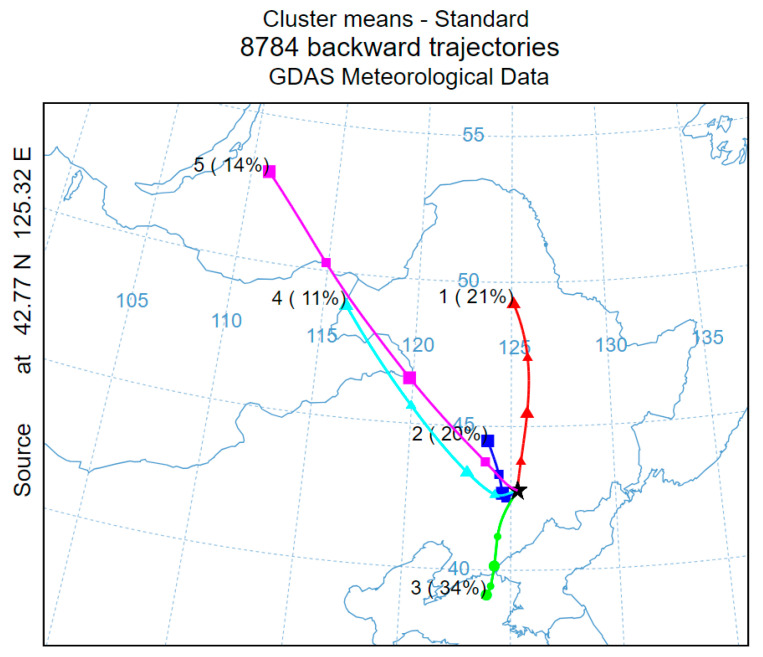
Backward trajectory clustering analysis in 2024.

Using Liaoyuan City (42.77° N, 125.32° E) as the receptor point for backward trajectory simulation, that is at the black pentagram shown in Figure 7, the simulation period covers the entirety of 2024, with an initial height set at 100 m and a 48 h backward trajectory calculated at 1 h intervals, resulting in a total of 8784 trajectories. The trajectories are clustered into five average transport pathways using the NCAR meteorinfo software version 5.7, as shown in Figure 7. The other results of HYSPLIT clustering are shown in Figures S4–S8. The results indicate that trajectories 2, 4, and 5 exhibit spatial consistency, with a cumulative contribution rate of 45%. These trajectories are influenced by northwesterly airflows, which act as carriers for pollutant transport, continuously delivering pollutants from the regions along the trajectories to Liaoyuan City, thereby significantly impacting the city’s O_3_ pollution levels. Among these, trajectory 2 has the highest proportion (20%) and the shortest transport distance, indicating lower wind speeds. Source analysis reveals that this trajectory originates from the urban agglomeration in the central–western part of Jilin Province. Similarly, trajectory 3 also belongs to the short-distance, low-wind-speed transport type. The cumulative contribution rate of these two short-distance transport trajectories is 54%. During the transport process, due to the slower movement of air masses, O_3_ and its precursors, as well as other photochemical products, have more time to accumulate, leading to their continuous buildup during transport [47]. When these air masses arrive in Liaoyuan City, they interact and combine with pollutants generated from local industrial production, vehicle emissions, and agricultural activities, creating synergistic effects that provide the material basis for high O_3_ concentrations [37]. Additionally, specific meteorological conditions further exacerbate the severity of O_3_ pollution in Liaoyuan City.

#### 3.2.3. Analysis of Heavy Pollution Episodes

**Figure 8:**
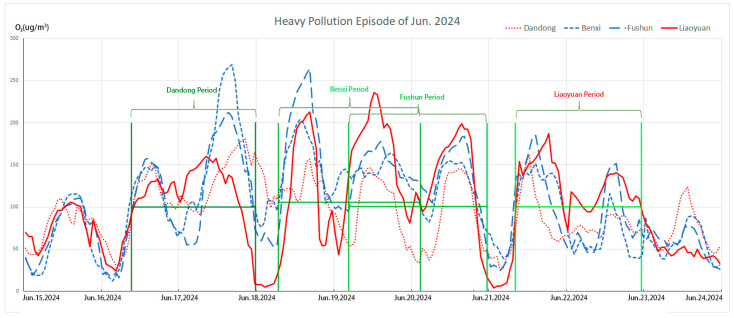
Period analysis of O_3_ heavy pollution episode in 2024.

**Figure 9:**
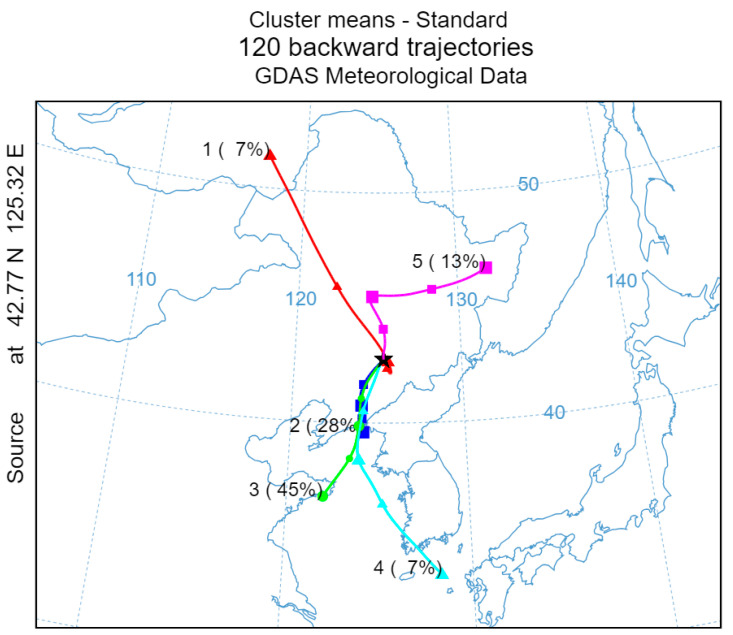
Backward trajectory clustering analysis of heavy pollution episode in 2024.

The monitoring data indicate that in June 2024, the proportion of days exceeding air quality standards in Liaoyuan City reached the highest level for the year, accounting for 36.7% of the month. Among these days, the number of days when O_3_ was identified as the primary pollutant was the highest. Therefore, this study selects a severe pollution period in mid-June (16–23 June) as the simulation period (we define this as more than 36 h of O_3_ heavy pollution even at night), as shown in Figure 8. Along path No. 3 in Figure 6 and path No. 2 in Figure 9, there is an obvious transportation route from the south to Liaoyuan City, which starts from East Sea, passing Dandong, Benxi City, and Fushun City. The Dandong period starts this heavy pollution episode in the morning on 16 June 2024, and the Liaoyuan period finishes the process at midnight on 23 June 2024. Relative research has found that a high O_3_ concentration will usually be accumulated in the mixed layer over the sea and will be transported to inland cities due to the land and sea breeze effect [48]. Thus, this should be the original source of this heavy pollution episode.

The backward trajectory clustering analysis is conducted to investigate the sources of pollution during heavy pollution days in Liaoyuan City, as illustrated in Figure 9. Using Liaoyuan City as the receptor site (the pentagram location in Figure 9, the backward trajectory simulations are initialized at an altitude of 100 m, with a time step of 1 h, and a total of 120 trajectories are obtained for a 48 h period. The cluster analysis identifies five major transport trajectories. Among them, trajectory 2 (28%) originates from the southwestern direction in Liaoning Province (Dandong–Benxi–Fushun region). Trajectory 3 (45%) originates from the eastern part of Shandong Province (Qingdao–Yantai region). Trajectory 4 (7%) originates from the southeast and has the longest transport distance. Collectively, these three transport pathways (trajectories 2, 3, and 4) account for 80% of the total, all covering maritime regions. The marine breeze facilitates the transfer of O_3_ from the oceanic atmosphere to the inland areas [48]. Simultaneously, these trajectories all traverse the central industrial belt of Liaoning Province [49], characterized by significant NO_X_ and VOC pollution. Consequently, these factors collectively contribute to the elevated O_3_ concentrations observed in Liaoyuan City. The synergistic effects of transboundary pollution transport and local emissions significantly contribute to the O_3_ pollution episode, resulting in four consecutive days of exceedances in Liaoyuan City. During this period, the daily maximum increase in O_3_ concentration is 208 μg/m^3^, with a peak concentration of 236 μg/m^3^ recorded on June 19.

#### 3.2.4. Analysis of Simulation of Air Pollution

**Figure 10:**
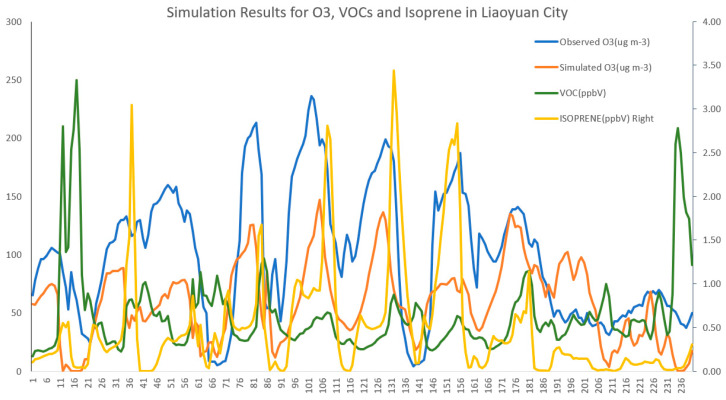
The simulation and verification results of air pollution during the heavy pollution episodes in Liaoyuan City in 2024.

**Figure 11:**
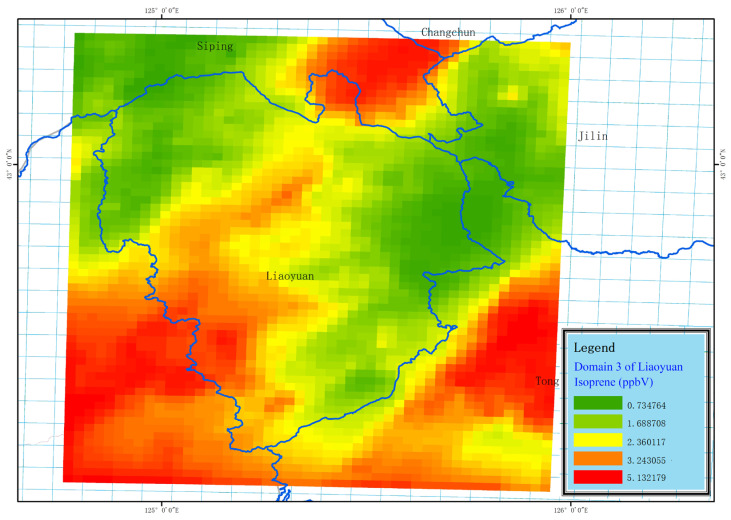
The spatial distribution of isoprene in Liaoyuan City during the heavy pollution episodes in 2024.

To study the relationship between the O_3_ and VOCs in the atmosphere of Liaoyuan City, we conduct spatiotemporal simulations of O_3_, VOCs, and isoprene, and the results are shown in Figure 10 and Figure 11. Moreover, to evaluate the simulation effect of the CMAQ model, we selected the pollutant-monitoring stations 2227A and 2228A within the d03 (Figure 1) area of Liaoyuan City for verification and analysis. The model simulation O_3_ concentration data in the CMAQ grid where the pollutant monitoring stations are located are extracted and compared with the monitoring data of the corresponding stations. Following the reference standards proposed by Boylan et al. [50], in this paper, the verification indicator R of the simulation data and monitoring data of the CMAQ model is 0.63, the MFB is −0.11, and the MFE is 0.17. There exists a 32% underestimation of the simulation’s O_3_ concentration due to MEIC emission inventory without considering natural emission sources (MEGAN). The simulation results are excellent. The results show that during the severe pollution period in 2024, the concentrations of O_3_ and VOCs present a significant negative correlation, with a correlation coefficient of −0.47. The correlation coefficient between the concentrations of O_3_ and isoprene is 0.27, and the ratio of pollutant VOCs to NOx is 13.26, indicating that Liaoyuan City is in a VOC-controlling area.

Through the ISAM source apportionment module of CMAQ, we conduct a source analysis of the regional contribution of O_3_ during the heavy pollution periods in Liaoyuan City in 2024. The results are shown in Table 6. Among the nine prefecture-level divisions in Jilin Province, the contribution of Changchun City sources to its O_3_ is the highest, but the proportion is only 5.24%. The contribution of regions other than the nine prefecture-level divisions within d03 (Figure 1) is 30.40%. The contribution rate outside the domain 3 region is 44.05%, among which the contribution of the southern region is 40.06% and that of the northern region is only 3.99%. It is proven that about 95% of the O_3_ pollution in Liaoyuan City originates from regional transport.

This research confirms what many previous atmospheric O_3_ studies have already found regarding O_3_ formation in the summer season, O_3_ precursors, and sources. The analysis of pollution sources during the heavy pollution period suggests that the pollution episode in Liaoyuan City was primarily driven by the combined effects of regional transport, local emissions, and meteorological conditions. During this period, the prevailing wind direction was southwesterly. Additionally, mid-June coincided with the peak period of fertilizer and pesticide application, increasing emissions from agricultural activities that contributed to O_3_ exceedances [51]. Furthermore, continuous emissions of NO_X_ from vehicle exhausts in urban areas exacerbated local pollution levels [52,53]. Unfavorable meteorological conditions, including strong solar radiation, high temperatures, and boundary layer compression, hindered pollutant dispersion and facilitated O_3_ accumulation [40]. These factors interacted, further deteriorating the air quality in Liaoyuan City and posing serious threats to urban environmental quality and public health [54].

## 4. Conclusions

Comparing the O_3_ pollution characteristics in Liaoyuan City with the surrounding cities, the O_3_ pollution in Liaoyuan City exhibits significant differences due to the influences of topography, industrial emissions, and variations in pollution control measures. The seasonal variation in O_3_ shows a high-value period from late spring to summer, characterized by a unimodal distribution peaking in May and June. This pattern is attributed to enhanced solar radiation and boundary layer elevation, which promote photochemical processes and drive O_3_ accumulation. In contrast, the lowest O_3_ concentrations occur in winter and early spring due to reduced photochemical reactivity under low temperatures. On a diurnal scale, O_3_ concentrations follow a distinct unimodal pattern, with peak values occurring in the afternoon, driven by the maximum intensity of solar radiation, which accelerates photolysis and facilitates the conversion of precursors into O_3_. Conversely, O_3_ concentrations remain low during the night-time and early morning, as nocturnal NO titration significantly reduces O_3_ levels.

A comparative analysis of three machine learning models indicates that the random forest model exhibits the best overall simulation performance, achieving an R^2^ value of 0.9043. Based on this model, this study comprehensively analyzes the factors influencing near-surface O_3_ concentrations in Liaoyuan City. The findings emphasize that precursor control remains the key strategy for mitigating O_3_ pollution, with a particular focus on the reduction in NO_X_ emissions [55]. Additionally, meteorological factors and underlying surface characteristics also play crucial roles in O_3_ concentration variations [56]. These insights provide valuable guidance for future O_3_ pollution control strategies.

By using backward trajectory clustering analysis, the ISAM source apportionment module of CMAQ, and spatiotemporal simulations, we find that O_3_ pollution in Liaoyuan City is significantly influenced by regional transport and local sources; the main reasons for this include meteorology and precursors. This analytical result is completely consistent with the previous results in this paper. Therefore, in the control of O_3_ pollution in Liaoyuan City, joint prevention and control must be carried out, with particular attention paid to pollution control under the condition of southerly wind weather. This emphasizes the importance of regional collaboration in addressing O_3_ pollution.

## Figures and Tables

**Table 1:** Parameterization scheme settings for WRF simulation.

Parameters	Settings
Microphysical Process Scheme	Thompson
Short-wave Radiation Scheme	Rapid Radiative Transfer Model
Long-wave Radiation Scheme	Rapid Radiative Transfer Model
Land surface Process Scheme	Noah Land Surface Model
Boundary Layer Scheme	YSU (Yonsei University)
Cumulus Parametric Scheme	Kain–Fritsch (New Eta)

**Table 2:** Formulas for evaluation indicators.

Indicators	Formulas
MFB	1N∑i=1Nmi−OiOi+mi2
MFE	1N∑i=1Nmi−OiOi+mi2
R	$\frac{\sum_{i=1}^{N}\left(m_{i}-\bar{m}\right)\left(O_{i}-\bar{O}\right)}{\sqrt{\sum_{i=1}^{N}{\left(m_{i}-\bar{m}\right)}^{2}}\sqrt{\sum_{i=1}^{N}{\left(O_{i}-\bar{O}\right)}^{2}}}$

**Table 3:** Correlations between pollutants and meteorological factors in Liaoyuan City for 2024.

Variables	PM_2.5_	PM_10_	SO_2_	NO_2_	O_3_	CO	T	P	WS
PM_2.5_	1.000								
PM_10_	0.900 **	1.000							
SO_2_	0.440 **	0.380 **	1.000						
NO_2_	0.470 **	0.410 **	0.290 **	1.000					
O_3_	−0.170 **	−0.160 **	−0.071 **	−0.540 **	1.000				
CO	0.630 **	0.530 **	0.260 **	0.630 **	−0.350 **	1.000			
T	−0.300 **	−0.270 **	−0.260 **	−0.260 **	0.210 **	−0.230 **	1.000		
P	−0.340 **	−0.310 **	−0.300 **	−0.290 **	0.230 **	−0.220 **	0.920 **	1.000	
WS	−0.028	0.0093	−0.030	−0.090 **	0.059 **	−0.099 **	0.240 **	0.0028	1.000

** *p* < 0.01.

**Table 4:** Proportion of primary pollutants in Liaoyuan City from 2021 to 2024.

Year	PM_2.5_ (%)	PM_10_ (%)	O_3_ (%)
2021	65.52	3.45	31.03
2022	51.30	0.00	48.70
2023	39.10	10.90	50.00
2024	42.10	5.30	52.60

**Table 5:** Feature importance ranking in the RF model.

ID	Variables	Categories	Value
1	NO_2_	Monitoring Pollutants	0.6579
2	Second Quarter	Temporal Variables	0.1105
3	Vegetation Coverage	Meteorological Variables	0.0598
4	Sea Surface Temperature	Meteorological Variables	0.0550
5	PM_2.5_	Monitoring Pollutants	0.0458
6	Water Vapor Content	Meteorological Variables	0.0399
7	Latent Heat	Meteorological Variables	0.0394
8	Downward Shortwave Radiation	Meteorological Variables	0.0355
9	CO	Monitoring Pollutants	0.0354
10	10 m Wind Speed	Meteorological Variables	0.0301
11	2 m Specific Humidity	Meteorological Variables	0.0256
12	Sensible Heat Flux	Meteorological Variables	0.0145
13	Ground Heat Flux	Meteorological Variables	0.0118
14	Surface Longwave Radiation	Meteorological Variables	0.0089
15	Outgoing Longwave Radiation	Meteorological Variables	0.0087

**Table 6:** ISAM result for Liaoyuan City in June 2024.

Area	Contribution Concentration (ug/m^3^)	Ratio (%)
Baicheng City	2.0	1.44
Baishan City	0.3	0.24
Changchun City	7.2	5.24
Jilin City	4.9	3.57
Liaoyuan City	7.0	5.05
Siping City	6.8	4.89
Songyuan City	3.0	2.15
Tonghua City	3.6	2.59
Yanbian	0.5	0.37
Southeast Area to domain 3	18.2	13.15
Southwest Plain Area to domain 3	12.6	9.10
Southwest Mountain Area to domain 3	24.6	17.81
North Area to domain 3	5.5	3.99
Other Area in domain 3	42.0	30.40

## Data Availability

All the data generated or analyzed during this study are included in this manuscript and the Supplementary Files.

## Supplementary Materials

The following supporting information can be downloaded at https://www.mdpi.com/article/10.3390/toxics13060500/s1, Figure S1: Random Forest Model Simulation Result; Figure S2: Artificial Neural Network Model Simulation Result; Figure S3: Support Vector Machine Model Simulation Result; Figure S4: Clusters number’s TSV curve for 2024 whole year in Liaoyuan; Figure S5: 5-Cluster means curve for 2024 whole year in Liaoyuan; Figure S6: Clusters number’s TSV curve for June of 2024 in Liaoyuan; Figure S7: 5-Cluster means curve for June of 2024 in Liaoyuan; Figure S8: 5-Cluster trajectories for June of 2024 in Liaoyuan.
