# Supplementary material for: Source Analysis of Ozone Pollution in Liaoyuan City’s Atmosphere Based on Machine Learning Models and HYSPLIT Clustering Method
Source: Toxics. 2025 Jun 13;13(6):500. doi: 10.3390/toxics13060500 (PMC12197544; doi:10.3390/toxics13060500)
Supplement: Supplementary file 1 [file toxics-13-00500-s001.zip › toxics-3626334-supplementary/toxics-3626334 supplementary word files-charts.pdf]

Optimum Setting of Grid Search

Number of Machine Learning	1000
Minimum fork sample size	2

Score of Cross-validation

	Cross Number	Score
0	1	0.8863
1	2	0.8832
2	3	0.8799
3	4	0.9078
4	5	0.8883

Model Accessment of Test Set

	MAE	MSE	R2
0	0.0385	0.0032	0.9043

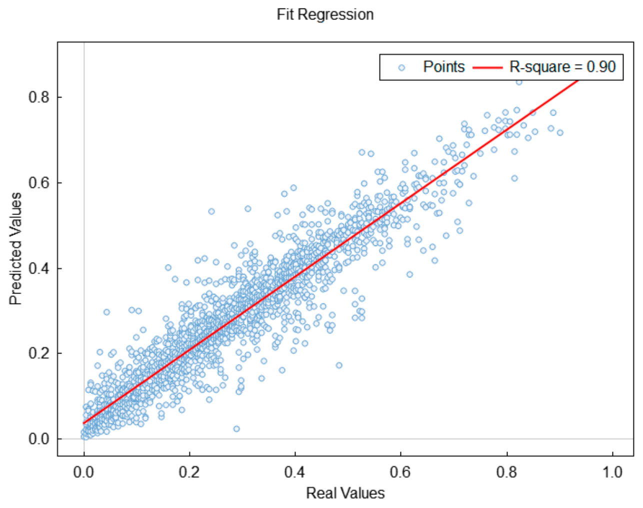

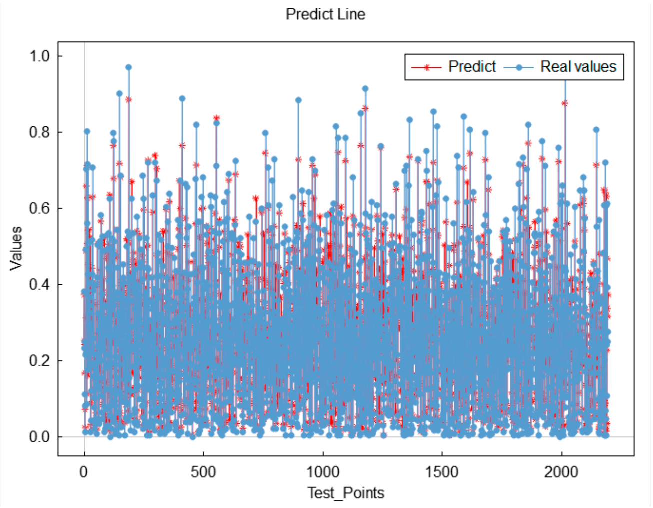

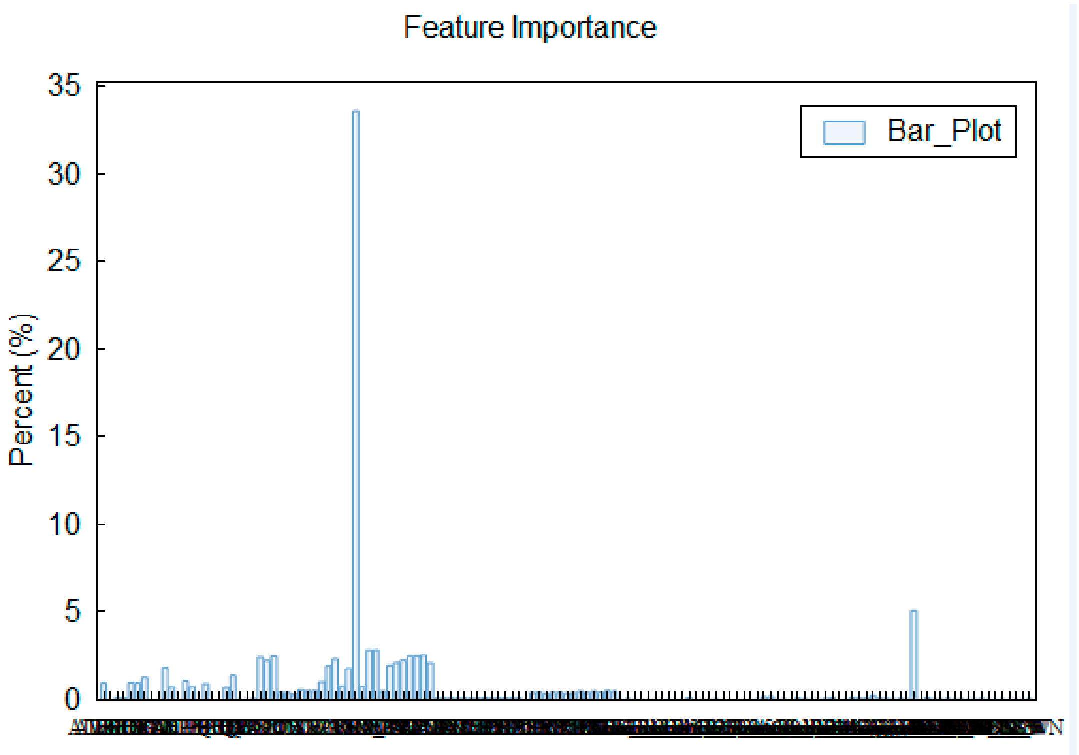

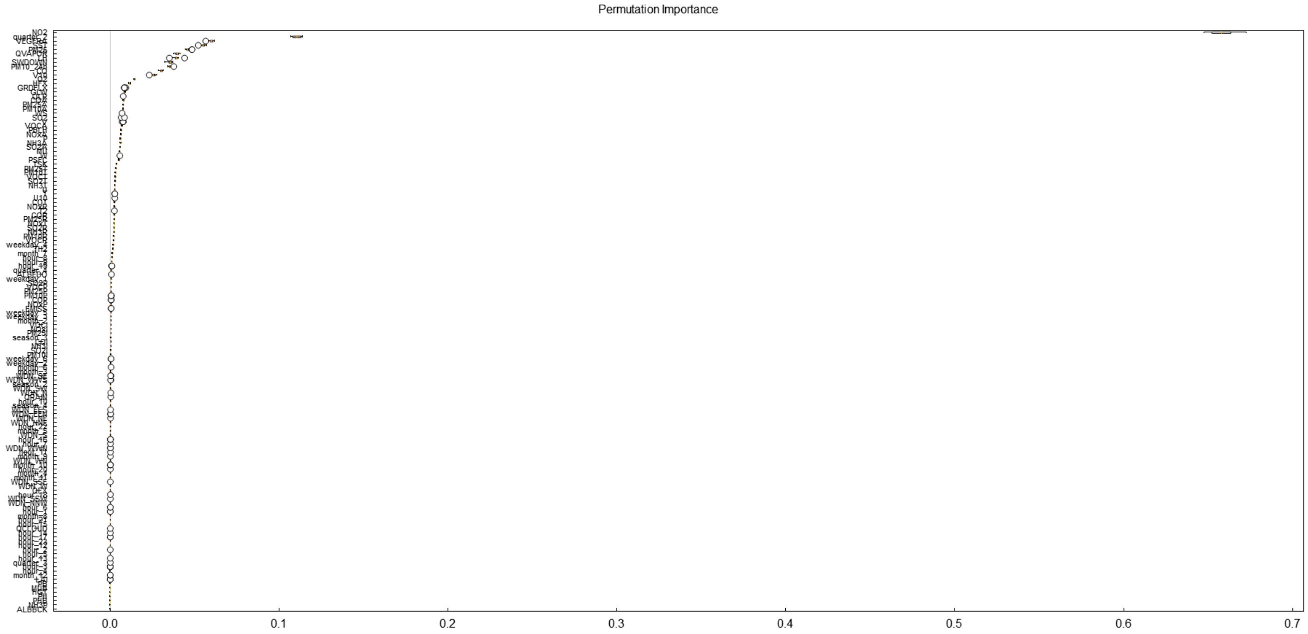

Figure S1. Random Forest Model Simulation Result

(results by DMSAS https://www.dmsas.cn)

Model Accessment of Test Set

	MAE	MSE	R2
0	0.0579	0.0059	0.8215

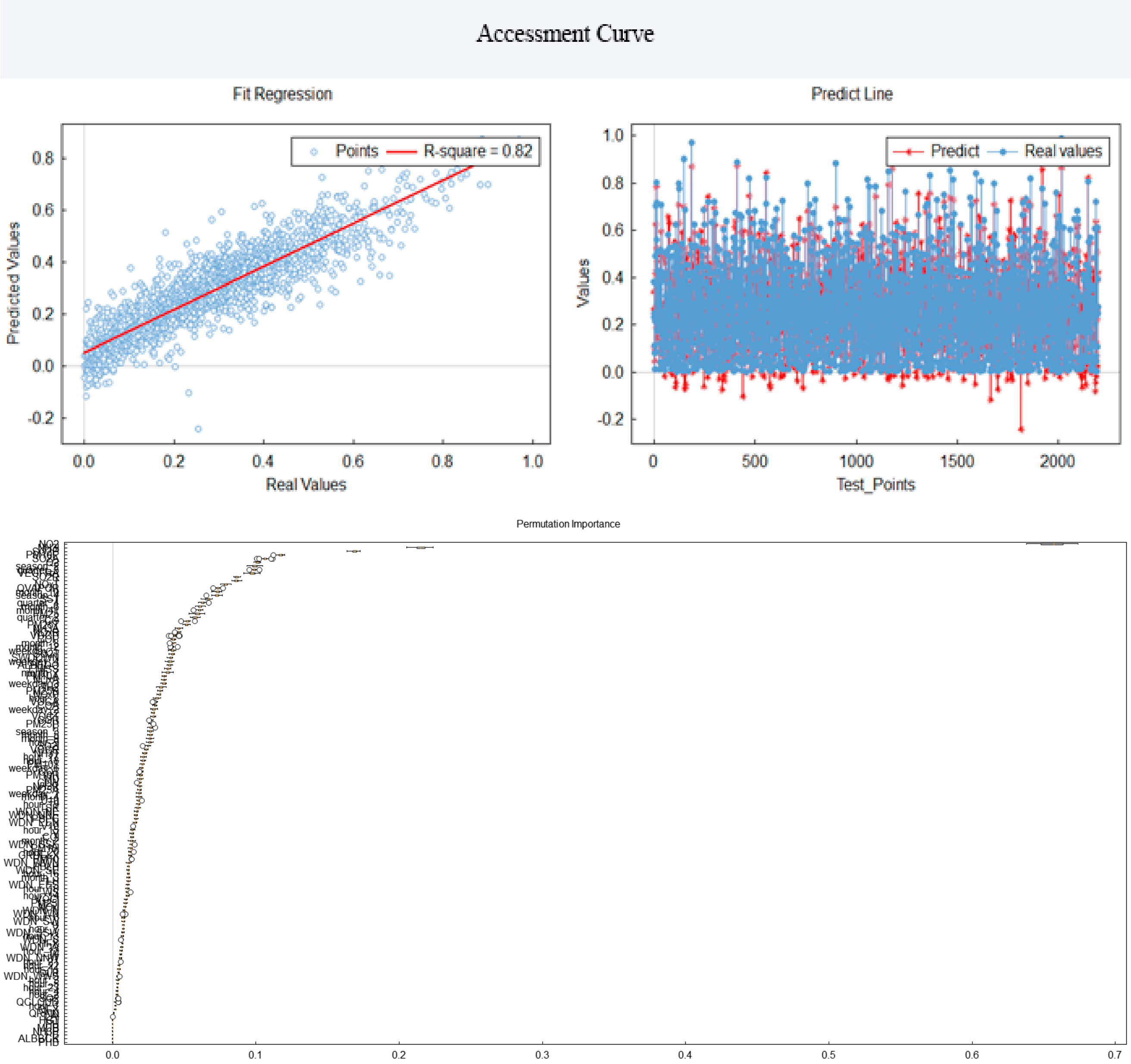

Figure S2. Artificial Neural Network Model Simulation Result
(results by DMSAS https://www.dmsas.cn)

Model Accessment of Test Set

	MAE	MSE	R2
0	0.0728	0.0091	0.7254

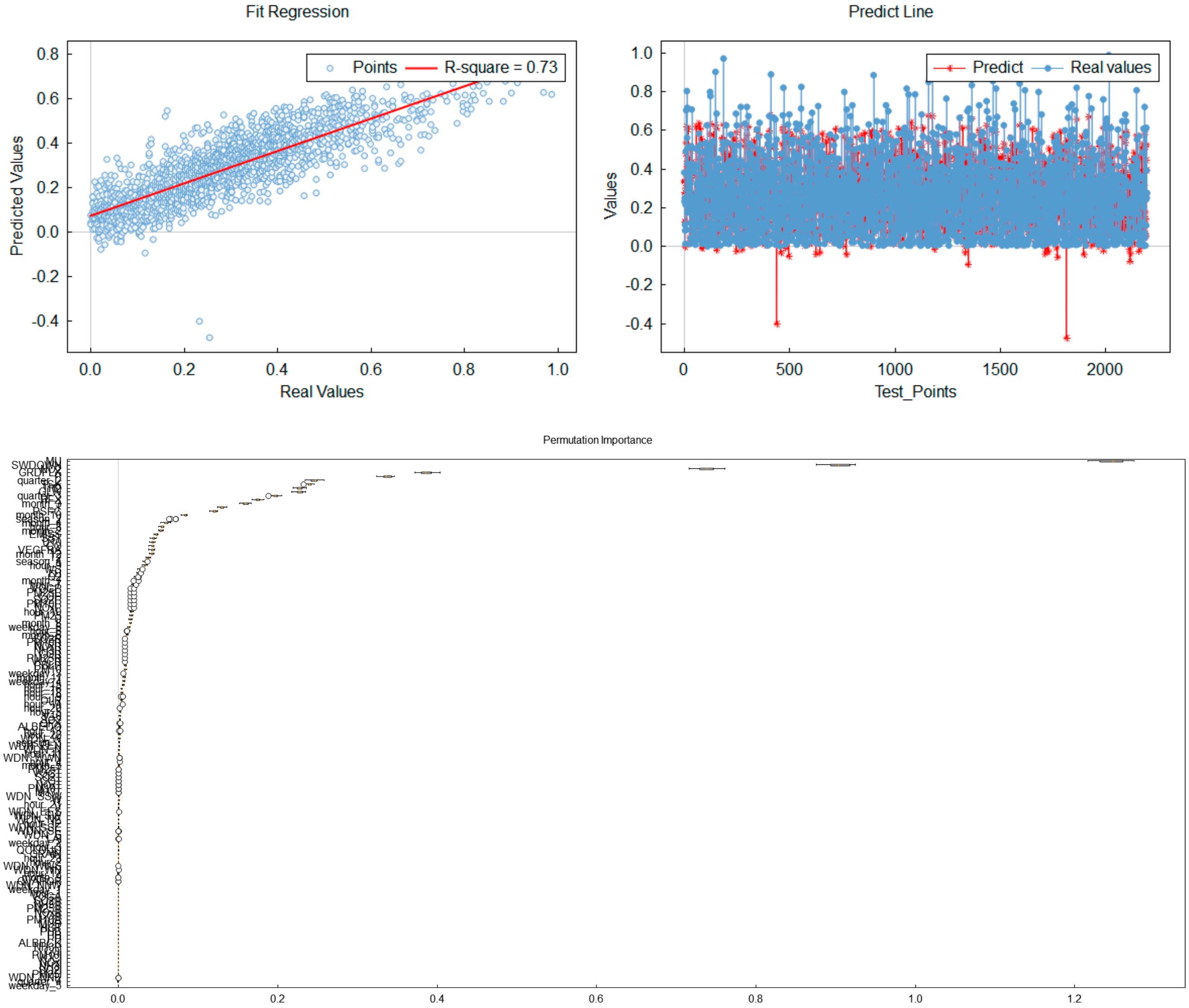

Figure S3. Support Vector Machine Model Simulation Result
(results by DMSAS https://www.dmsas.cn)

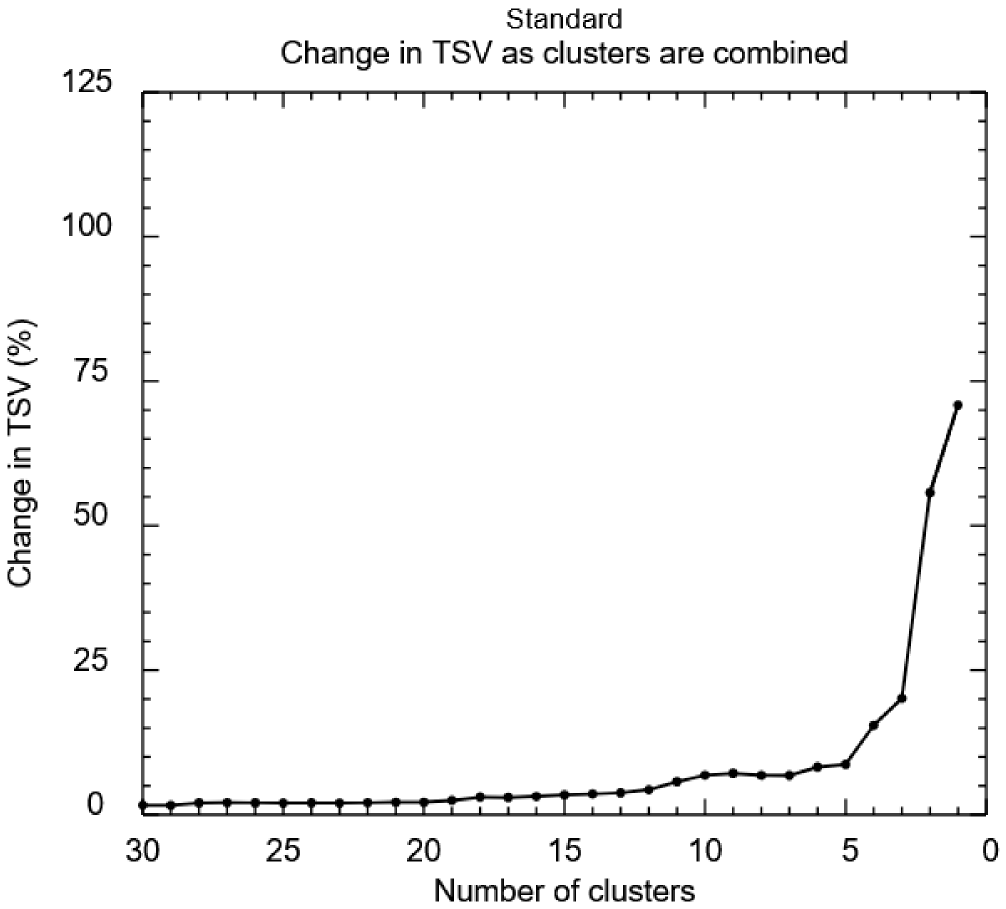

Figure S4. Clusters number's TSV curve for 2024 whole year in Liaoyuan

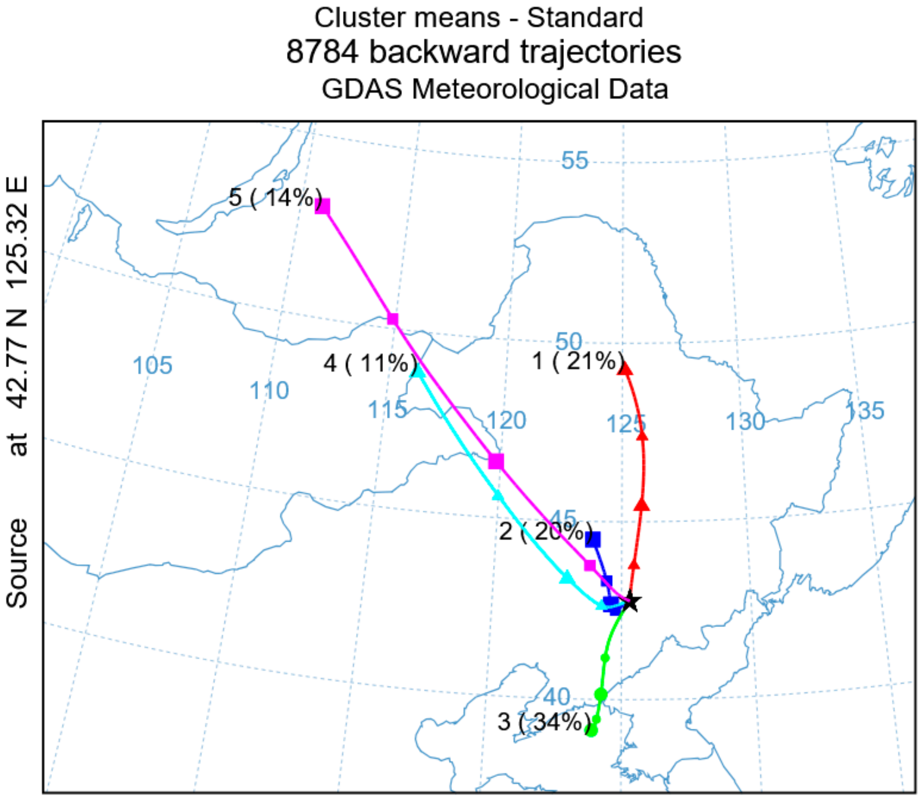

Figure S5. 5-Cluster means curve for 2024 whole year in Liaoyuan

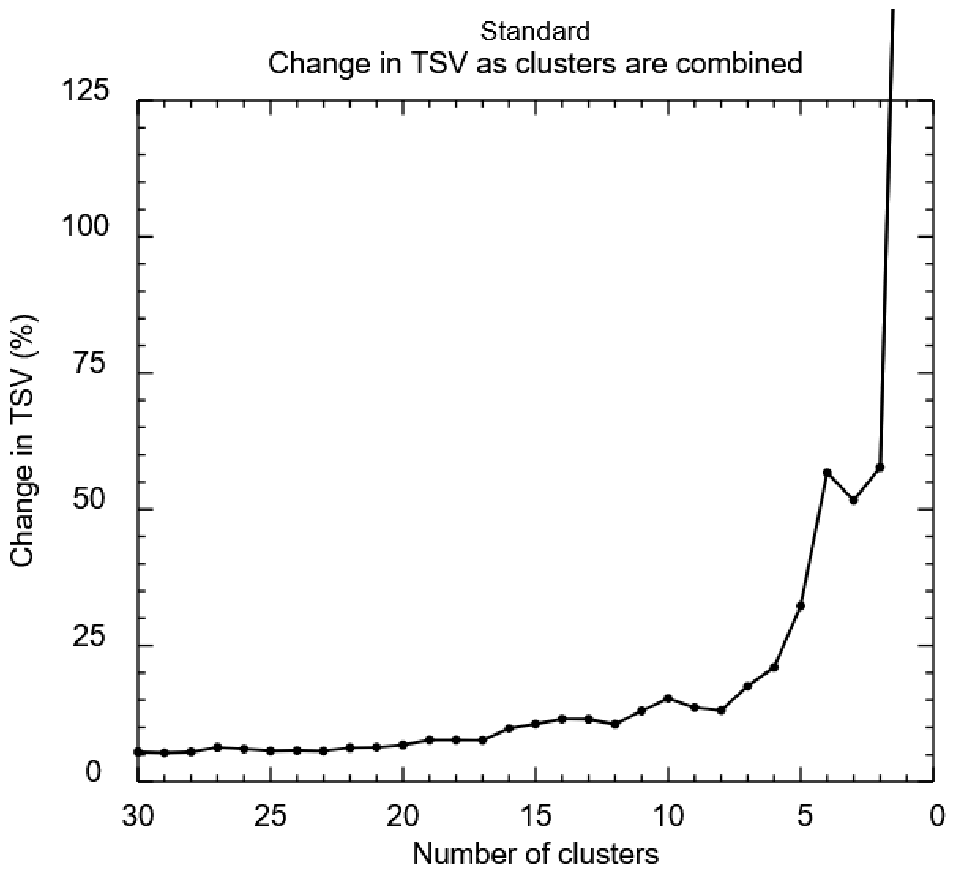

Figure S6. Clusters number's TSV curve for June of 2024 in Liaoyuan

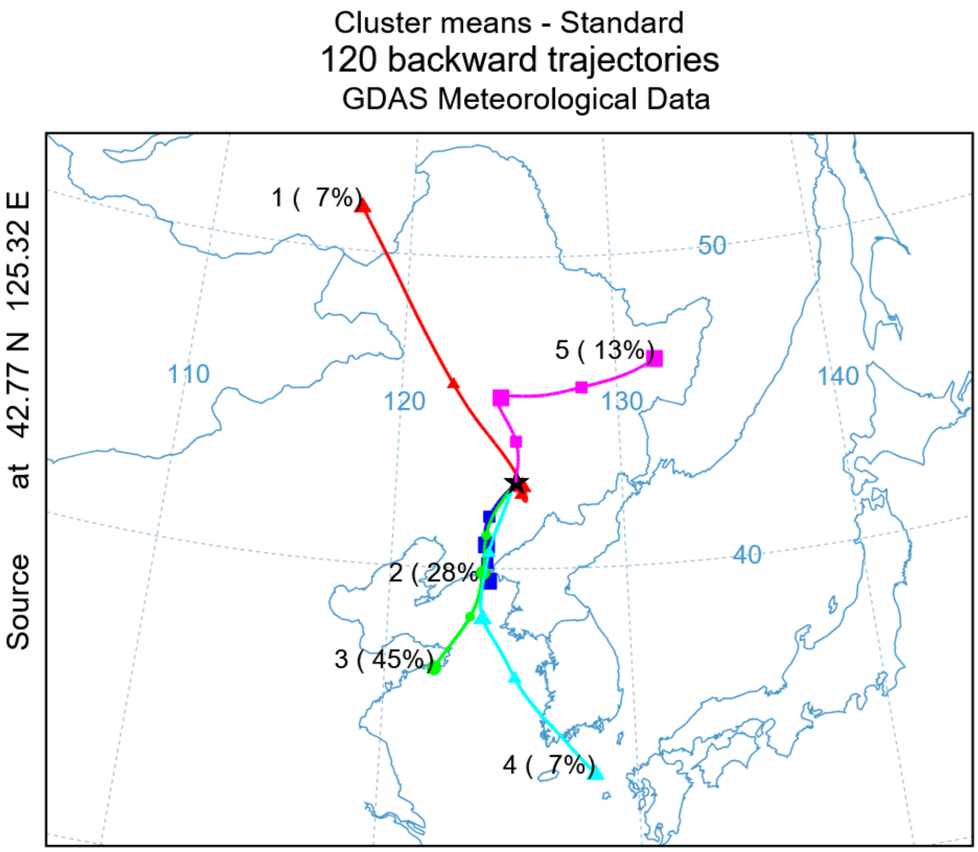

Figure S7. 5-Cluster means curve for June of 2024 in Liaoyuan

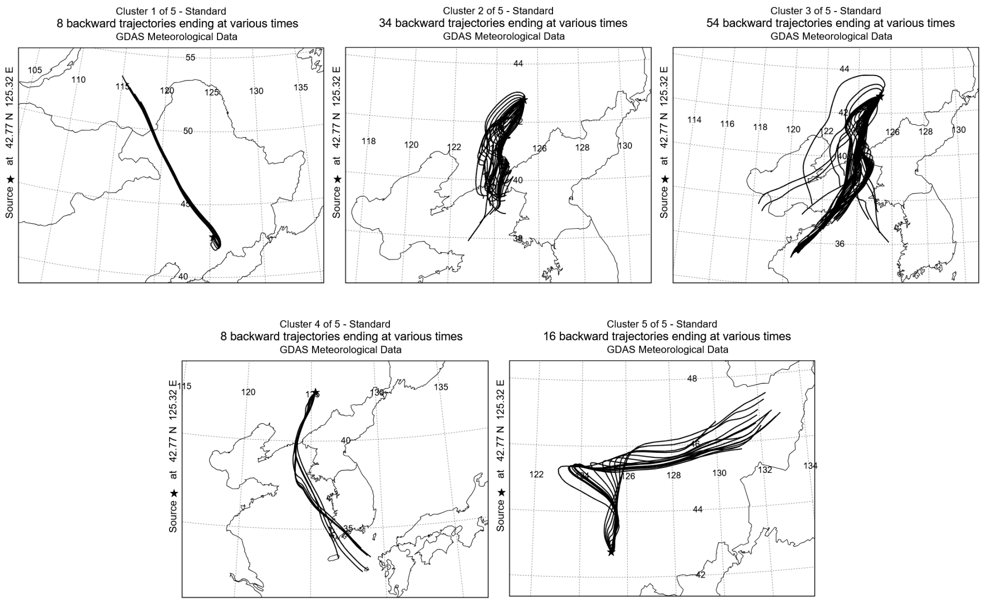

Figure S8. 5-Cluster trajectories for June of 2024 in Liaoyuan
